# Looking Beyond the Bleed: A Case Report

**DOI:** 10.7759/cureus.36897

**Published:** 2023-03-30

**Authors:** Rachel Noto, Angela Creditt

**Affiliations:** 1 Emergency Medicine, Virginia Commonwealth University, Richmond, USA

**Keywords:** altered mental state, puerperium, intracerebral hemorrhage, headache disorders, cerebral venous and dural sinus thrombosis

## Abstract

Cerebral venous thrombosis (CVT) can be one of the most challenging diagnoses to make in a patient with an undifferentiated headache. A missed diagnosis of the condition can lead to catastrophic consequences, as seen in the case described here. There needs to be a high index of clinical suspicion for CVT as the diagnosis involves imaging modalities that are not frequently used in the emergency setting. This case report demonstrates how the classic avenues of headache workup can miss this diagnosis. It also illustrates how delayed diagnosis can present in the extremis and have unsalvageable outcomes.

## Introduction

Available data suggest that cerebral venous thrombosis (CVT) is a rare diagnosis with an annual incidence ranging from 0.22 to 1.6 per 100,000 people [1,2]. It is a disease that affects women more commonly than men at a ratio of 3:1. Some cite an incidence rate as high as 2.78 per 100,000 in women between the ages of 31 and 50 years [1]. This skewed sex ratio is thought to be associated with the use of oral contraceptives, pregnancy, and the puerperium period [3,4]. Other risk factors for developing CVT include hypercoagulable states such as obesity, cancer, and genetic thrombophilia. In younger people, diabetes mellitus and diabetic ketoacidosis have a strong association with CVT. The most common symptoms at presentation are headache, seizures, or focal neurologic deficit. CVT is thought to arise from thrombosis of cerebral sinuses causing restrictive outflow, resulting in increased intracranial hypertension and, therefore, a headache. The second stage of the disease involves increasing venous and capillary pressure, resulting in the breakdown of the blood-brain barrier, leading to brain edema and venous infarction. Venous infarction is uniquely often hemorrhagic [5]. An intracerebral hemorrhage is present on imaging at the time of diagnosis in approximately 40% of cases [5]. Altered mental status or coma can also result from CVT. One in seven patients is comatose on admission [6]. In this report, we discuss a case of CVT presenting with intracranial hemorrhage.

## Case presentation

A 34-year-old female presented to the emergency department (ED) via emergency medical services (EMS) for the evaluation of her altered mental status. Per EMS, the patient had been recently admitted to an outside hospital where she had been diagnosed with headache, new-onset diabetes mellitus, and idiopathic intracranial hypertension. The family had reported to EMS that the patient's last known well time (LKWT) had been the previous night. Additional history from the patient was significantly limited due to the patient's mental status change.

Collateral history was obtained through the patient's family member. The patient had a past medical history of asthma, obesity, and tubal ligation. She had been feeling unwell and had been recently admitted to an outside hospital five days ago for a severe headache. During that admission, she had undergone a head CT, brain MRI, ocular ultrasound, and lumbar puncture (LP), all of which had been unremarkable except for a finding of elevated pressure on LP. The patient continued to complain of a severe headache during her inpatient stay, which was reportedly attributed to her LP the day prior. Her condition had improved slightly and she had been discharged home on two new medications. The family reported that she had continued to feel unwell following discharge, and she had vomited the previous night. This morning she was taken to the emergency department after she was found lying on the ground confused with bowel and bladder incontinence.

On arrival at the ED, additional information was gathered from medical records during her recent hospital stay. It was reported that the patient had been admitted after presenting complaints of a headache beginning three weeks prior to the presentation. Her headache had been noted to be worse with standing, improved with lying down, and associated with nausea. She also had elevated glucose and new bilateral pedal edema on a physical exam. The initial head CT had been reported to be normal. She had been further evaluated with an MRI brain. The MRI had been normal, and hence the patient had undergone an LP. The LP results had been significant for elevated opening pressure and normal fluid analysis. Following the procedure, she had a worsening headache which had been attributed to the LP. The patient had been discharged home with normal mentation and started on acetazolamide and metformin for the new diagnoses.

The initial evaluation of the patient's second visit to the emergency department was significant for confusion, mild hypertension, and mild tachycardia. She was afebrile and oriented only to herself. A focal neurologic assessment could not be completed as the patient was not following commands. Point of care glucose was in the 500s on arrival. Given the patient's altered mental status, multiple labs and imaging modalities were obtained including a venous blood gas (Table 1) [7], complete blood count (Table 2), comprehensive metabolic panel, troponin, thyroid level, ammonia, lactate (Table 3), and coagulation studies (Table 4) [8]. Electrocardiogram (EKG), serum drug screen, urine analysis, and pregnancy test were also performed.

**Table 1:** Venous blood gas pH: potential of hydrogen; pCO_2_: partial pressure of carbon dioxide; pO_2_: partial pressure of oxygen; HCO_3_: bicarbonate; BE: base excess

Venous blood gas				
		Patient value	Reference range	Unit
pH		7.26	7.30-7.43	
pCO_2_		33	38-58	mmHg
pO_2_		40	19-65	mmHg
HCO_3_ calc		14.8	22-30	mmol/L
BE calc		12.6	1.9-4.5	mmol/L

**Table 2:** Complete blood count WBC: white blood cell; RBC: red blood cell; HCT: hematocrit; PLT: platelet

Complete blood count				
		Patient value	Reference range	Unit
WBC		18.7	4.0-10	10^9^/L
RBC		5.64	4.2-5.9	10^12^/L
Hemoglobin		13.7	12.0-16.0	g/dL
HCT		44.1	36-47	%
PLT		481	150-350	10^9^/L

**Table 3:** Comprehensive metabolic panel, troponin, TSH, ammonia, and lactate BUN: blood urea nitrogen; AST: aspartate aminotransferase; ALT: alanine transaminase; ALP: alkaline phosphatase; TSH: thyroid-stimulating hormone

Comprehensive metabolic panel				
		Patient value	Reference range	Unit
Sodium		141	136-145	mmol/L
Potassium		4.6	3.5-5.0	mmol/L
Chloride		106	98-106	mmol/L
Carbon dioxide		14	23-28	mmol/L
Glucose		523	70-100	mg/dL
BUN		16	8-20	mg/dL
Creatinine		0.99	0.7-1.3	mg/dL
AST		15	0-35	unit(s)/L
ALT		11	0-35	unit(s)/L
ALP		153	36-92	unit(s)/L
Total bilirubin		0.5	0.3-1.2	mg/dL
Conjugated bilirubin		0.2	0-0.3	mg/dL
Total protein		9.4	6.0-7.8	g/dL
Albumin		5.1	3.5-5.5	g/dL
Globulin		4.3	2.5-3.5	g/dL
Calcium		11.3	9-10.5	mg/dL
Magnesium		2.7	1.5-2.4	mg/dL
Troponin		<0.03	<0.05	ng/mL
TSH		0.59	0.5-5.0	uU/mL
Ammonia		31	23-47	umol/L
Lactate		9.3	0.67-1.8	mmol/L

**Table 4:** Coagulation factors PT: prothrombin time; aPTT: activated partial thromboplastin clotting time

Coagulation factors			
	Value	Reference range	Unit
PT	16	11-13	Seconds
aPTT	23	25-35	Seconds
D-dimer	15.74	<0.05	mg/L

The EKG interpretation was as follows: nonischemic, normal axis, sinus tachycardia, and normal PR intervals without ST changes. Drug screen, salicylate level, acetaminophen level, urine analysis, and pregnancy tests were negative.

**Figure 1:**
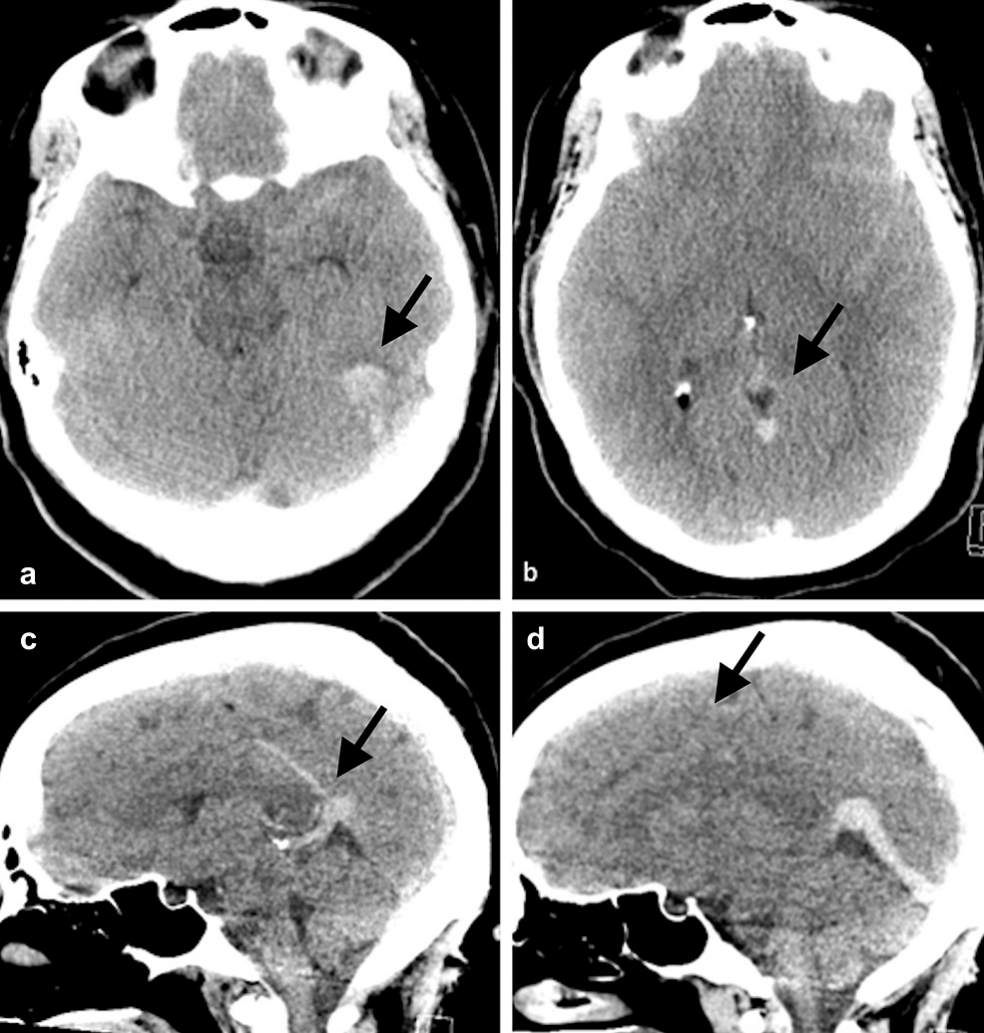
CT head without contrast Small intraparenchymal hematoma in the left temporal lobe (a) and a small amount of subarachnoid hemorrhage in the left cerebral hemisphere (b). Diffuse dural sinuses involving the superior sagittal sinus, transverse sinuses bilaterally, and inferior sagittal sinus (c). Multiple thrombosed cortical veins and evidence of deep venous thrombosis involving the vein of Gallen and straight sinus to the torcula (d). Diffuse sulcal effacement and ventricular compression, in keeping with increased intracranial pressure. There is no transtentorial or tonsillar herniation CT: computed tomography

The patient then emergently underwent a non-contrast CT head (Figure 1).

**Figure 2:**
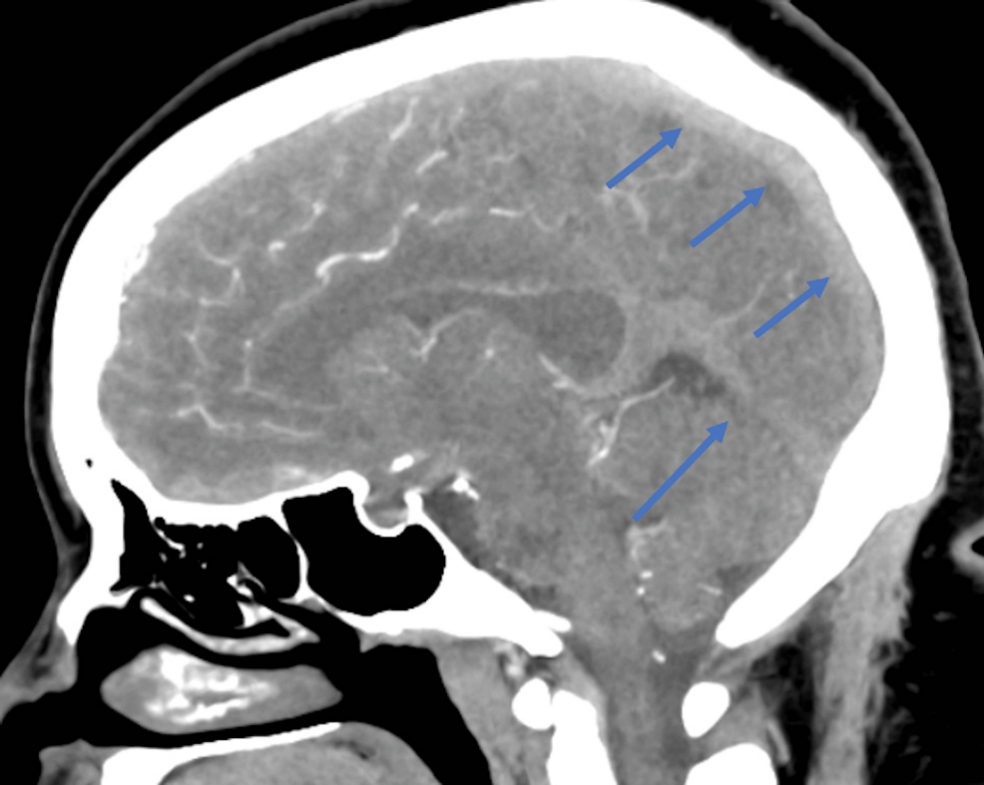
CT angiogram of the head and neck There is evidence of extensive dural sinus and deep venous thrombosis (blue arrows), although the study is not optimized to evaluate venous structures. There is a lack of evidence of arterial stenosis, aneurysm, or arterial venous malformation CT: computed tomography

Given the results of the CT head, further imaging was obtained with a CT angiogram head and neck (Figure 2).

An emergent neurosurgery consult was placed for the evaluation of extensive dural venous thrombosis, cerebral venous thrombosis, and associated intraparenchymal and subarachnoid hemorrhage. Heparin and clevidipine infusions were started. Upon reassessment, after the imaging was complete, the patient became minimally responsive with somnolence and an inability to protect the airway. She was subsequently intubated using etomidate and rocuronium. Fentanyl and propofol infusions were started for sedation.

The patient was quickly admitted to the neurosurgery ICU and taken to the operating room for a diagnostic cerebral angiogram and venogram. This confirmed the suspicion of extensive dural venous sinus thrombosis and cerebral venous thrombosis not amenable to thrombectomy or recanalization. Overnight, the patient decompensated, requiring blood pressure support and desmopressin for the development of diabetes insipidus. Throughout the night, the clinical picture continued to decline and eventually, the patient passed away later that evening.

## Discussion

In this case, it is clear that the delay in diagnosis of CVT led to a fatal outcome despite the patient’s extensive headache workup. An underlying pathology was only considered after further decline occurred and intracranial hemorrhage was discovered on the CT head despite the risk factors of obesity and diabetes. Due to the rare nature of this disease, this is not an uncommon occurrence. The most common presenting symptoms of CVT are headache, increased intracranial pressure, focal neurologic deficits, and seizures. The clinical manifestations are highly variable and mimic other disease forms, which often leads to a delay in diagnosis, with a median delay of symptom onset to diagnosis of seven days [6]. The main imaging modality in the diagnosis of CVT includes CT or MRI venography, a study not routinely obtained in the emergency setting [5]. Approximately 30-50% of CVT patients present with some form of intracerebral hemorrhage [5]. This suggests that venous infarction and the breakdown of the blood-brain barrier may correlate with advanced disease. The mainstay of treatment is heparin and endovascular treatment, with recanalization in approximately 90% of patients, of which there is an 80% recovery rate without long-term symptoms [5]. Mortality in CVT has been declining over the years; while rates were as high as 50% previously, now it is thought to be between 5 and 10% [5]. Death in these cases often occurs due to large venous infarcts or transtentorial herniation, which was the case in the patient discussed here.

## Conclusions

It is important to consider CVT in any patient presenting with an undifferentiated headache. A missed diagnosis of this condition can lead to catastrophic consequences. It is especially important to keep CVT on the differential in any patient with any hypercoagulable state, as this has proven to be the main risk factor for developing this disease. This includes genetic, hormonal, infectious, and metabolic causes. There needs to be a high index of clinical suspicion for CVT as the diagnosis involves imaging modalities that are not frequently used in the emergency setting.
